# Altered Ca^2+ ^homeostasis in polymorphonuclear leukocytes from chronic myeloid leukaemia patients

**DOI:** 10.1186/1476-4598-5-65

**Published:** 2006-11-27

**Authors:** Chetana M Revankar, Suresh H Advani, Nishigandha R Naik

**Affiliations:** 1 Biochemistry and Cell Biology, Cancer Research Institute, ACTREC, TMC, Navi Mumbai 410210, India; 2 Tata Memorial Hospital, Tata Memorial Centre, Parel, Mumbai 400 012, India; 3 Dept of Oncology, Lombardi Comprehensive Cancer Center, Georgetown University Medical Center, W412 Research Building, 3970 Reservior Road NW, Washington DC, USA; 4 Director, Medical Oncology, Jaslok Hospital and Research Centre, 15, Dr. G. Deshmukh Marg, Peddar Road, Mumbai 400 026, India

## Abstract

**Background:**

In polymorphonuclear leukocytes (PMNL), mobilization of calcium ions is one of the early events triggered by binding of chemoattractant to its receptors. Besides chemotaxis, a variety of other functional responses are dependent on calcium ion mobilization. PMNL from chronic myeloid leukaemia (CML) patients that were morphologically indistinguishable from normal PMNL were found to be defective in various functions stimulated by a chemoattractant – fMLP. To study the mechanism underlying defective functions in CML PMNL, we studied calcium mobilization in CML PMNL in response to two different classical chemoattractants, fMLP and C5a.

**Results:**

Release of calcium estimated by flow cytometry and spectrofluorimetry using fluo-3 as an indicator showed that the [Ca^2+^]_i _levels were lower in CML PMNL as compared to those in normal PMNL. But, both normal and CML PMNL showed maximum [Ca^2+^]_i _in response to fMLP and C5a at 10 sec and 30 sec, respectively. Spectrofluorimetric analysis of the total calcium release in chemoattractant treated PMNL indicated more and faster efflux of [Ca^2+^]_i _in CML PMNL as compared to normal PMNL.

**Conclusion:**

Fine-tuning of Ca^2+ ^homeostasis was altered in CML PMNL. The altered Ca^2+ ^homeostasis may contribute to the defective functions of CML PMNL.

## Background

In polymorphonuclear leukocytes (PMNL), changes in intracellular calcium, i.e. [Ca^2+^]_i _are associated with multiple cellular events, including activation of cellular kinases and phosphatases, degranulation, phagosome-lysosome fusion, regulation of cytoskeleton binding proteins, transcriptional control and modulation of surface receptors [1]. Migration of leukocytes through the extracellular matrix to the site of action is the first step in host defence and role of calcium in this process is well reviewed by Maxfield [2]. Although no stable [Ca^2+^]_i _gradients were detected in migrating human PMNL, a transient global increase in [Ca^2+^]_i _was found to be important for chemotaxis [3]. PMNL migration can be induced by binding of chemoattractants to their receptors present on PMNL surface. The classical chemoattractants for PMNL are n-formyl peptides that are analogous to bacterial secretion [4] and anaphylatoxin C5a, which is formed upon complement activation [5]. Specific receptors for n-formyl peptides and C5a are present on PMNL and they share common structural motifs [6]. Mobilization of [Ca^2+^]_i _is one of the early events triggered by binding of a chemoattractant to its receptor.

Chronic myeloid leukaemia (CML) is a clonal, pluoripotent stem cell disorder characterized by the occurrence of Philadelphia chromosome (Ph^1^) and presence of a large number of mature and immature myeloid cells in the peripheral blood [7]. Earlier work from our laboratory has shown that PMNL from CML patients were defective in actin dependent functions such as chemotaxis, degranulation, endocytosis, etc. [8-12]. Chemotaxis was found to be defective in all the phases of the disease [9]. Calcium plays a central role in these functions. Calcium regulates cell motility by regulating polymerization of actin – one of the major motile machinery proteins in PMNL. Increased [Ca^2+^]_i _levels lead to fragmentation of actin network by disrupting the cross bridges of actin network. Increased [Ca^2+^]_i _levels cause fragmentation of F-actin by activation of actin severing and capping proteins such as gelsolin and macrophage capping protein [13]. Fibroblasts transfected with gelsolin, a calcium activated actin severing and capping protein, display increased motility [14]. In view of the role of calcium in various motility related events, the present studies are aimed to study mobilization of Ca^2+ ^in CML PMNL. Mobilization of Ca^2+ ^by fMLP and C5a was studied in these cells. PMNL from healthy normal individuals were used as control. We found that fine-tuning of Ca^2+ ^homeostasis in CML PMNL was altered as compared to that in normal PMNL.

## Results

### Measurement of [Ca^2+^]_i _by flow cytometry

#### Basal [Ca^2+^]_i _levels in PMNL

**Figure 1:**
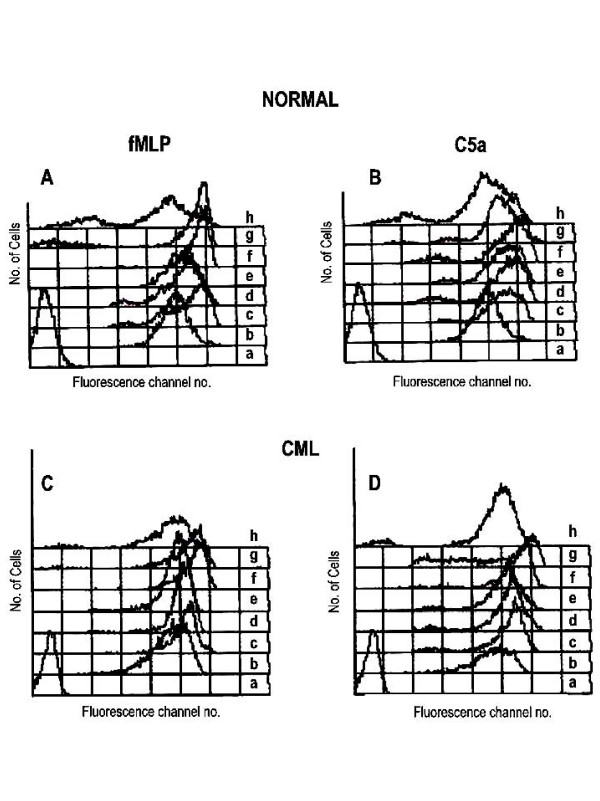
**Flow cytometric histogram overlay of fluo-3 loaded PMNL**. Representative plots of fMLP or C5a stimulated PMNL from normal donors (A and B) and CML patients (C and D). (a) isotype, (b) unstimulated, stimulated with fMLP or C5a for (c) 10 sec, (d) 30 sec, (e) 60 sec, (f) calcium ionophore A23187, (g) EGTA and (h) MnCl_2_. X-axis indicates fluorescence channel No. and Y-axis indicates No. of cells.

Fluo-3 loaded normal PMNL showed a broad bell shaped plot, indicating considerable variation in the basal [Ca^2+^]_i _levels of the normal PMNL population (Fig. 1). Fluo-3 loaded unstimulated CML PMNL showed a broad plot with a long tail near Y-axis, containing 9–10% of the population. Thus, 10% of the population had very low levels of [Ca^2+^]_i_. Comparison between the two populations showed that basal levels of [Ca^2+^]_i _in CML PMNL were lower and showed more variation. However, this difference was not statistically significant (Table 1).

**Table 1:** Flow cytometric estimation of [Ca^2+^]_i _in fMLP stimulated normal and CML PMNL.

Stimulant	n	Nil (Basal level)	fMLP					
Time			10 s	30 s	60 s	Calcium ionophore	EGTA	MnCl_2_

Normal	25	291.24 ± 53.95	472.15^# ^± 106.93	466.93^# ^± 158.45	339.34^# ^± 69.58	728.07^# ^± 155.05	601.76^# ^± 155.55	193.14^$ ^± 39.20
CML	27	236.69 ± 22.33	412.31^# ^± 106.52	384.76^# ^± 88.21	248.15^# ^±64.75	610.28^# ^± 158.6	601.84^# ^± 164.83	240.32^# ^± 75.77

All values are average ± SEM

n = Number of samples.

# Significantly higher value over the respective basal level, p < 0.05.

$ Significantly lower value as compared to the basal level, p < 0.05.

#### Stimulation with fMLP

**Figure 2:**
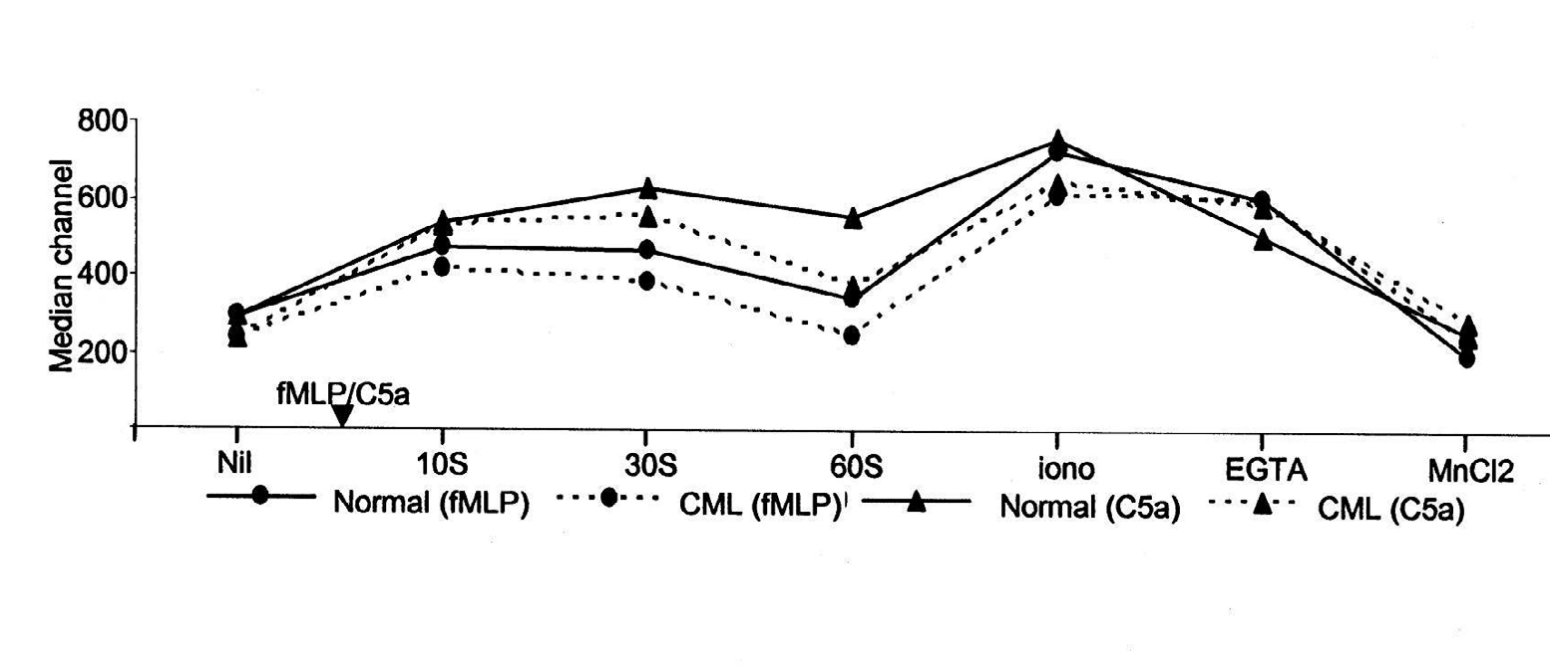
**Flow cytometric estimation of [Ca^2+^]_i _levels in PMNL**. Fluo-3 loaded CML and normal PMNL were stimulated with fMLP (10^-8 ^M) or C5a (10^-9 ^M) followed by treatment with calciumionophore A23187, EGTA, and MnCl_2_. X-axis indicates the treatment given to the cells and Y-axis indicates average median fluorescence channel values.

In fMLP stimulated normal PMNL, plots were negatively skewed and broader. Hence the heterogeneity in the PMNL population with respect to [Ca^2+^]_i _levels increased. On stimulation, the peaks shifted to right (Fig. 1A; c, d, e) leading to a significant increase in [Ca^2+^]_i _levels at 10 sec, 30 sec, and 60 sec (Table 1 and Fig. 2), maximum increase in [Ca^2+^]_i _being at 10 sec. On further treatment of these cells with calcium ionophore A23187, [Ca^2+^]_i _levels increased significantly (Fig. 1A; f, Table 1), but heterogeneity in PMNL with respect to [Ca^2+^]_i _decreased. Addition of EGTA resulted in building up of a small peak towards extreme left on the X-axis indicating that only 8–10% of the total population, was sensitive to EGTA (Fig. 1A; g). But the resulting decrease in total [Ca^2+^]_i _levels was considerable. On further addition of MnCl_2_, [Ca^2+^]_i _levels decreased significantly and were below the basal levels (Fig. 1A; h, Table 1). The PMNL population was extremely heterogeneous as far as sensitivity to quenching of calcium by MnCl_2 _was considered.

**Figure 3:**
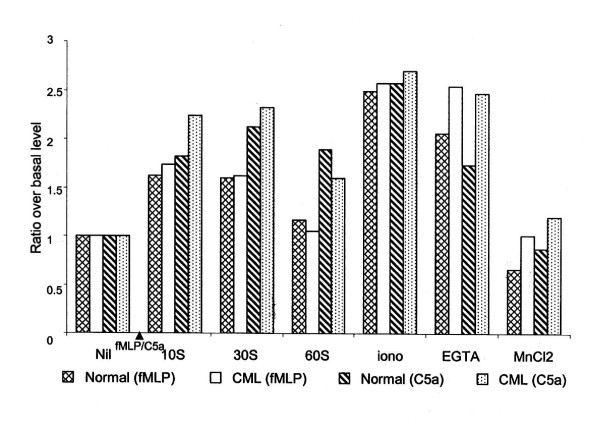
**Flow cytometric estimation of extent of stimulation of [Ca^2+^]_i _in PMNL**. Fluo-3 loaded CML and normal PMNL were stimulated with fMLP (10^-8 ^M) or C5a (10^-9 ^M), followed by treatment with calcium ionophore A23187, EGTA and MnCl_2_. X-axis indicates the treatment given to the cells and Y-axis indicates ratios of average [Ca^2+^]_i _levels in stimulated cells to the [Ca^2+^]_i _levels in unstimulated cells, i.e. basal level.

To compare the extent of stimulation in PMNL, the ratio of [Ca^2+^]_i _levels before and after fMLP stimulation were calculated. In normal PMNL, 10 sec of fMLP treatment resulted in 1.25 to 4.4 times (mean ± SEM = 1.6 ± 0.21) increase in [Ca^2+^]_i _over the basal levels. Further treatment with ionophore increased the mean ratio to 2.5 ± 0.36. Subsequent addition of EGTA and MnCl_2 _decreased [Ca^2+^]_i _levels to 21% and 64% of the maximum levels, respectively; ultimately quenching down the fluorescence to about 30% lower than the basal levels (Fig. 3).

CML PMNL showed narrowing of the PMNL peak after fMLP stimulation, indicating decrease in heterogeneity in the population with respect to [Ca^2+^]_i _levels (Fig. 1C; b, c, d, e). At all the time points studied after fMLP stimulation, levels of [Ca^2+^]_i _were significantly higher than the basal [Ca^2+^]_i _levels (Table 1, Fig. 2). The peak levels were seen at 10 sec. On further treatment of these cells with calcium ionophore A23187, the [Ca^2+^]_i _levels increased further, but shape of the peak was unaltered (Fig. 1C; f). On addition of EGTA, the total [Ca^2+^]_i _content in the population was not altered considerably (Fig. 1C; g). On further addition of MnCl_2_, the peak width increased, the peak shifted towards Y-axis and an additional very small peak appeared adjacent to Y-axis (Fig. 1C; h). Thus, the PMNL population was extremely heterogeneous as far as sensitivity to quenching of calcium by MnCl_2 _was considered. The ratio of maximum fluorescence in fMLP stimulated cells ranged from 1.42 to 5.62. On addition of ionophore the mean ratio increased to 2.6. Addition of EGTA led to 10% decrease in the fluorescence intensity where as MnCl_2 _resulted in a statistically significant 60% decrease as compared to the maximum fluorescence intensity. Thus, EGTA and MnCl_2 _together brought down the fluorescence to the basal level (Fig. 3).

Both, normal and CML PMNL, showed significantly higher [Ca^2+^]_i _levels on fMLP stimulation as compared to that in the respective unstimulated PMNL(Fig. 1A and 1C). But on fMLP stimulation, the heterogeneity with respect to [Ca^2+^]_i _levels was lower in CML PMNL than in normal PMNL. The [Ca^2+^]_i _levels both before and after fMLP stimulation were lower in CML than in normal PMNL. However, this difference was not statistically significant. In fMLP stimulated CML PMNL, the drop in levels of [Ca^2+^]_i _at 60 sec was rapid and more as compared to that in normal PMNL. This was evident from the higher ratio over the basal level at 60 sec in normal PMNL as compared to that in CML PMNL. On the addition of calcium ionophore A23187, fMLP stimulated CML PMNL showed lower levels of [Ca^2+^]_i _as compared to normal PMNL. But the extent of [Ca^2+^]_i _mobilization was higher in CML as reflected in the ratios (Fig. 3). Though the extent of [Ca^2+^]_i _stimulation with fMLP and ionophore was higher in CML PMNL than that in normal, it was not statistically significant.

Quenching of [Ca^2+^]_i _with EGTA showed considerable decrease in [Ca^2+^]_i _levels in normal PMNL but not in CML PMNL. On further quenching of [Ca^2+^]_i _by MnCl_2_, the [Ca^2+^]_i _levels were maintained above the basal levels in CML PMNL whereas, in normal PMNL these were lower than the basal levels (Table 1). Significant quenching of [Ca^2+^]_i _is seen on addition of MnCl_2 _in both the populations. When ratios of these EGTA and MnCl_2 _treated normal and CML populations were compared they were significantly higher in CML PMNL than the respective normal PMNL. Thus, it shows that though the levels of [Ca^2+^]_i _and quenching of [Ca^2+^]_i _were always lower in CML PMNL, the extent of stimulation, i.e. the ratios of these [Ca^2+^]_i _levels to the basal levels were maintained at higher levels in CML PMNL as compared to that in normal PMNL.

#### Stimulation with C5a

C5a stimulated normal PMNL showed broad negatively skewed peaks (Fig. 1B; c, d, e). The [Ca^2+^]_i _levels were significantly higher than the basal [Ca^2+^]_i _levels. The maximum increase was at 30 sec after C5a stimulation (Fig. 2 and Table 2). Treatment of these cells with calcium ionophore A23187 resulted into two peaks. A major peak was seen with a modal channel shifted to right as compared to that seen at 30 sec (Fig. 1B; f), indicating a further increase in [Ca^2+^]_i _levels. A small population of PMNL that formed a minor peak was probably non-respondent to the ionophore treatment. The addition of EGTA resulted in a significant shift of the modal channel of the major peak to the left and its broadening (Fig. 1B; g). This showed increased heterogeneity in normal PMNL with respect to quenching of [Ca^2+^]_i _by EGTA.

**Table 2:** Flow cytometric estimation of [Ca^2+^]_i _in C5a stimulated normal and CML PMNL.

Stimulant	n	Nil (Basal level)	C5a					
Time			10 s	30 s	60 s	Calcium ionophore	EGTA	MnCl_2_

Normal	20	293.94 ± 46.48	535.84^# ^± 102.31	626.19^# ^± 166.79	556.62^# ^± 114.85	757.45^# ^± 106.95	510.05^# ^± 96.67	256.52^$ ^± 51.12
CML	21	237.05 ± 36.44	532.58^# ^± 108.54	552.20^# ^± 94.31	378.60 ± 70.98	642.25^# ^±162.82	586.81 ± 157.76	284.99^# ^± 103.32

All values are average ± SEM.

n = Number of samples.

# Significantly higher value over the respective basal level, p < 0.05.

$ Significantly lower value as compared to the basal level, p < 0.05.

Though after EGTA treatment, [Ca^2+^]_i _levels reduced significantly than that in ionophore treated PMNL, they remained at a significantly higher level than the basal [Ca^2+^]_i _levels (Table 2). On further addition of MnCl_2_, the major peak broadened further and both the peaks shifted towards Y-axis showing heterogeneity in the PMNL population as far as sensitivity to quenching of calcium by MnCl_2 _was considered (Fig. 1B; h). After MnCl_2 _treatment the [Ca^2+^]_i _levels reached below the basal [Ca^2+^]_i _levels and these were significantly lower than that in ionophore treated PMNL. To compare the extent of stimulation in PMNL, the ratio of [Ca^2+^]_i _levels before and after C5a stimulation was calculated. In normal PMNL, it ranged from 1.2 to 2.89. On further treatment with ionophore though the mean ratio increased to 2.57 ± 0.25, this increase was not statistically significant. Further additions of EGTA and MnCl_2 _led to 14% and 55% quenching of fluorescence. This decrease in fluorescence was statistically significant (Fig. 3 and Table 2).

C5a stimulated CML PMNL showed negatively skewed peaks that were shifted towards right (Fig. 1D; c, d, e). These increases in [Ca^2+^]_i _levels after C5a stimulation were significantly higher than the basal [Ca^2+^]_i _levels. Maximum increase in the [Ca^2+^]_i _levels was seen at 30 sec after C5a stimulation (Fig. 2 and Table 2). On treatment of these cells with calcium ionophore A23187 [Ca^2+^]_i _levels increased further. The addition of EGTA resulted in the building up of an extended tail on the left side of the peak indicating heterogeneity in PMNL population as far as sensitivity to quenching of calcium by EGTA was considered. About 20% of the total population was lying in this tail and hence the decrease in total [Ca^2+^]_i _levels was considerable (Table 2). On further addition of MnCl_2_, a major bell shaped peak along with a minor peak towards extreme left was seen (Fig. 1D; g). Thus, PMNL population was extremely heterogeneous as far as sensitivity to quenching of calcium by MnCl_2 _was considered. This further reduced [Ca^2+^]_i _levels to considerably lower levels (Table 2). In CML PMNL the ratio over basal level ranged from 1.45 to 3.77. Though addition of ionophore increased the mean ratio to 2.71 ± 0.23, it was not statistically significant. In contrast to this, sequential addition of EGTA and MnCl_2 _decreased the fluorescence intensity significantly, by 15% and 58%, respectively (Fig. 3).

Both normal and CML PMNL showed significantly higher [Ca^2+^]_i _levels on C5a stimulation as compared to the respective basal [Ca^2+^]_i _levels. But the heterogeneity with respect to [Ca^2+^]_i _levels was higher in CML PMNL. Though at 10 sec after C5a stimulation the levels of [Ca^2+^]_i_, were comparable in normal and CML PMNL, at later time points these were higher in normal PMNL. However, these differences were not statistically significant. Maximum stimulation was seen at 30 sec in both the populations. In CML PMNL the [Ca^2+^]_i _levels increased by 2.33 times whereas in normal PMNL it increased by 2.13 times the basal level (Table 2 and Fig. 3). The extents of stimulation were higher in C5a stimulated CML PMNL at 10 sec and 30 sec as compared to normal PMNL. At 60 sec, a steep decrease in [Ca^2+^]_i _levels was seen in CML PMNL while in normal PMNL it was gradual. This resulted in reversing the scenario (Fig. 3). On addition of calcium ionophore A23187 subsequent to C5a stimulation, CML PMNL showed a lower amount of [Ca^2+^]_i _as compared to normal PMNL (Table 2). But extent of stimulation was higher in CML PMNL than that in normal PMNL. However, this was not statistically significant. Similarly differences in the decrease in the [Ca^2+^]_i _by EGTA and MnCl_2 _in normal and CML PMNL were non-significant (Table 2, Fig. 3).

In both CML and normal PMNL, the [Ca^2+^]_i _levels in C5a stimulated PMNL were higher than fMLP stimulated PMNL. The maximum [Ca^2+^]_i _levels were seen at 10 sec and 30 sec after fMLP and C5a stimulation, respectively (Fig. 2). When the maximum [Ca^2+^]_i _levels obtained by treatment of PMNL with calcium ionophore A23187 and minimum [Ca^2+^]_i _levels obtained by quenching with EGTA and MnCl_2_, in fMLP and C5a stimulated normal and CML PMNL were compared, they were not significantly different. Thus, though levels of [Ca^2+^]_i _seen in fMLP and C5a stimulated normal and CML PMNL differed, the behavioural pattern, i.e. mode of alterations in [Ca^2+^]_i _levels with respect to time, was similar in both the populations.

#### Measurement of total Ca^2+ ^by spectrofluorimetry

Mobilization of [Ca^2+^]_i _eventually leads to efflux of [Ca^2+^]_i_. Therefore, to estimate total mobilization of Ca^2+ ^we have quantitated Ca^2+ ^levels in fluo-3 loaded normal and CML PMNL by spectrofluorimetry.

#### Basal Ca^2+ ^levels in PMNL

The basal levels of Ca^2+ ^in normal PMNL ranged from 224 to 386 nM whereas in CML PMNL it ranged from 175 to 342 nM. As seen in flow cytometric assay the basal levels of Ca^2+ ^were lower in CML PMNL as compared to that in normal PMNL (Table 3). However, this difference was statistically non-significant.

**Table 3:** Spectrofluorimetric estimation of Ca^2+ ^in fMLP stimulated normal and CML PMNL.

Stimulant	n	Nil (Basal level)	fMLP				
Time			10 s	30 s	60 s	EGTA	MnCl_2_

Normal	25	304.74 ± 75.78	467.34^# ^± 97.99	373.88^# ^± 56.58	275.06^# ^± 24.46	339.60^# ^± 31.70	135.39^$ ^± 21.03
CML	27	285.48 ± 34.13	692.65^# ^± 95.92	488.17^# ^± 89.92	334.66 ± 79.09	366.30^# ^± 51.64	132.14^$ ^± 19.60

All values are average ± SEM, in nM.

n = Number of samples.

# Significantly higher value over the respective basal level, p < 0.05.

$ Significantly lower value as compared to the basal level, p < 0.05.

#### Stimulation with fMLP

**Figure 4:**
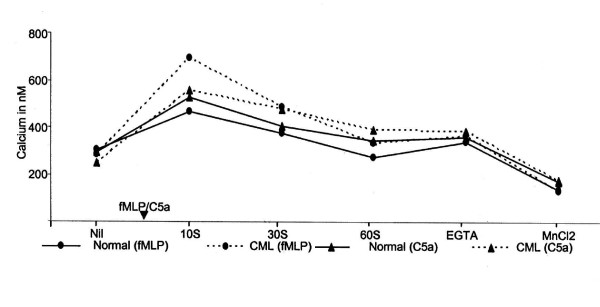
**Total Ca^2+ ^levels estimated by spectrofluorimetric assay**. CML and normal PMNL were stimulated with fMLP (10^-8 ^M) or C5a (10^-9 ^M) and the changes in calcium levels measured at 10 sec, 30 sec and 60 sec. The PMNL were further treated with 10 μM calcium ionophore A23187, EGTA, and MnCl_2_. The fluorescence of the fluo-3 loaded PMNL was measured in arbitrary fluorescence units. X-axis indicates the treatment given to the cells and Y-axis indicates the average of the total calcium in nM

**Figure 5:**
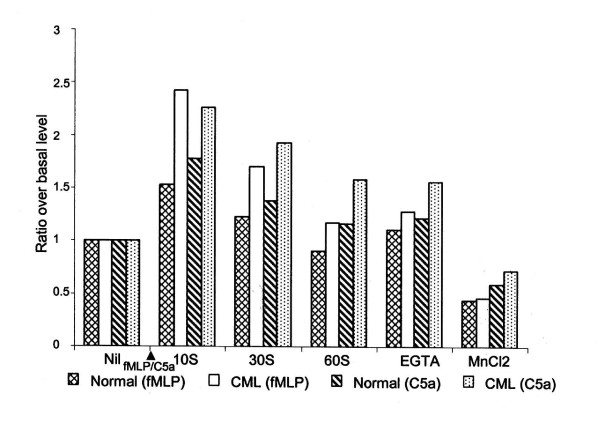
**Spectrofluorimetric estimation of extent of stimulation of [Ca^2+^]_i _in PMNL**. Extent of stimulation of calcium over the basal level in fMLP or C5a stimulated CML and normal PMNL. Ratios of the total calcium in PMNL after stimulation or addition of EGTA and MnCl_2 _to the basal level were calculated. X-axis indicates the treatment given to the cells and Y-axis indicates average of ratios.

On fMLP stimulation of normal PMNL, the total amount of Ca^2+ ^increased significantly at 10 sec and 30 sec as compared to the basal levels; whereas at 60 sec it was significantly lower as compared to the basal level (Table 3). Peak Ca^2+ ^levels were seen at 10 sec (Fig. 4). To compare the extent of stimulation in PMNL, the ratio of Ca^2+ ^levels before and after fMLP stimulation were calculated. In normal PMNL, this ratio ranged from 1.2 to 2.09 (Fig. 5). On addition of EGTA little quenching of the fluo-3 fluorescence was seen as compared to the fluorescence intensity of the calcium ionophore A23187 treated cells. However, the levels of Ca^2+ ^were still significantly higher as compared to the basal levels. The ratio of the two was 1.11 ± 0.23. On addition of MnCl_2_, a significant quenching of fluorescence occurred, bringing down the ratio to 0.44 ± 0.11 (Fig. 5).

In CML PMNL, the total amount of Ca^2+ ^was significantly higher at 10 sec and 30 sec after fMLP stimulation as compared to basal levels. Whereas at 60 sec, Ca^2+ ^levels were higher than the basal level but it was statistically non-significant. Peak Ca^2+ ^levels were seen at 10 sec (Table 3 and Fig. 4). This was also evident from the ratio over the basal level at different time points after fMLP stimulation, which ranged from 1.45 to 2.70. Similar to normal PMNL, about 30% quenching of fluo-3 was seen on the addition of EGTA as compared to the [Ca^2+^]_i _levels achieved after ionophore addition. The Ca^2+ ^levels were 1.28 ± 0.33 times higher than the basal levels. This difference between Ca^2+ ^levels was statistically significant. On addition of MnCl_2 _the Ca^2+ ^levels decreased significantly, lowering down the ratio to 0.46 ± 0.06 (Fig. 5).

In fMLP stimulated CML PMNL, the Ca^2+ ^levels as well as extent of stimulation were higher than those in normal PMNL (Fig. 5). However, the differences between the two populations were statistically non-significant. The drop in Ca^2+ ^levels after reaching the peak levels was higher in CML PMNL as compared to that in normal PMNL. But, since the Ca^2+ ^levels had reached much higher in CML PMNL as compared to normal PMNL these remained higher than the basal levels for a longer time.

#### Stimulation with C5a

In normal PMNL, total amount of Ca^2+ ^was significantly higher at all the time points after C5a stimulation as compared to the basal levels, the peak Ca^2+ ^levels being at 10 sec (Table 4 and Fig. 4). The ratio of Ca^2+ ^levels of C5a stimulated normal PMNL over basal Ca^2+ ^levels ranged from 1.25 to 2.56. On addition of EGTA, little quenching of the fluo-3 fluorescence was seen as compared to the fluorescence intensity of the calcium ionophore A23187 treated cells. Though the levels of Ca^2+ ^were higher as compared to the basal levels, they were statistically non-significant.  The ratio of the two was 1.22 ± 0.33 (Fig. 5). On addition of MnCl_2_, a significant quenching of fluorescence occurred lowering down the ratio to 0.59 ± 0.12 (Fig. 5). 

**Table 4:** Spectrofluorimetric estimation of Ca^2+ ^in C5a stimulated normal and CML PMNL.

Stimulant	n	Nil (Basal level)	C5a				
Time			10 s	30 s	60 s	EGTA	MnCl_2_

Normal	20	294.81 ± 34.62	524.9^# ^± 83.11	406.23^# ^± 3.89	343.22^# ^± 38.27	358.91 ± 45.82	174.62^$ ^± 42.88
CML	21	246.86 ± 22.13	558.49^# ^± 87.80	478.11^# ^± 3.44	390.70^# ^± 67.81	387.28^# ^± 42.06	179.59^$ ^± 25.64

All values are average ± SEM, in nM.

n = Number of samples.

# Significantly higher value over the respective basal level, p < 0.05.

$ Significantly lower value as compared to the basal level, p < 0.05.

Similar to normal PMNL, CML PMNL showed significantly higher Ca^2+ ^levels on C5a stimulation that peaked at 10 sec (Table 4 and Fig. 4). In these cells, the ratio over basal Ca^2+ ^levels ranged from 1.69 to 2.75. Quenching of fluo-3 was seen on addition of EGTA as compared to the fluorescence intensity of the ionophore treated cells. The Ca^2+ ^levels were 1.57 ± 0.33 times higher than the basal levels. However, this difference was not statistically significant. On further addition of MnCl_2_, the Ca^2+ ^levels decreased significantly, thereby reducing the ratio to 0.73 ± 0.12 (Fig. 5). Thus on C5a stimulation, both, CML and normal PMNL showed significant increase in the Ca^2+ ^levels reaching maximum at 10 sec (Table 4). The concentration of Ca^2+ ^and extent of stimulation were higher in CML PMNL as compared to that in normal PMNL (Fig. 4). But these differences were not statistically significant. The drop in Ca^2+ ^concentrations after reaching the peak stimulation was rapid and more in CML PMNL as compared to that in normal PMNL (Fig. 4).

In normal PMNL, at all the time points after stimulation the levels of Ca^2+ ^were higher in C5a stimulated PMNL than that in fMLP stimulated PMNL (Fig. 4 and Table 4). In CML PMNL, at 10 sec and 30 sec after stimulation Ca^2+ ^levels were higher in fMLP stimulated PMNL than those in C5a stimulated PMNL. At 60 sec calcium levels were higher in C5a stimulated CML PMNL as compared to fMLP stimulated CML PMNL (Fig. 4 and Table 4). Thus, alterations were seen in the levels and time kinetics of Ca^2+ ^mobilization in CML PMNL in response to fMLP and C5a stimulation. Levels of Ca^2+ ^reached after addition of EGTA and MnCl_2 _were slightly higher in fMLP stimulated PMNL as compared to that in C5a stimulated PMNL (Table 3 and 4). However, this difference was statistically non-significant. Thus, flow cytometric and spectrofluorimetric estimation of Ca^2+^, indicated a rapid and greater efflux of [Ca^2+^]_i _in CML PMNL as compared to that in normal PMNL. But in spite of more efflux of [Ca^2+^]_i_, the [Ca^2+^]_i _levels in CML PMNL after stimulation were maintained above the basal level. Whereas in normal PMNL, the [Ca^2+^]_i _levels after stimulation either dropped down below basal level or were slightly higher. In summary, the fine-tuning of Ca^2+ ^homeostasis is altered in CML PMNL as compared to that in normal PMNL.

## Discussion

Binding of the chemoattractants to their receptors on PMNL transmits signals across the plasma membrane, initiating phosphophorylation cascade and changes in [Ca^2+^]_i _concentration, that are crucial to cell activation. Both the release of calcium from the intracellular stores and the influx of calcium from the extracellular space contribute to the rise in [Ca^2+^]_i _levels. This [Ca^2+^]_i _in turn regulates chemoattractant receptor availability in PMNL [15].

**Figure 6:**
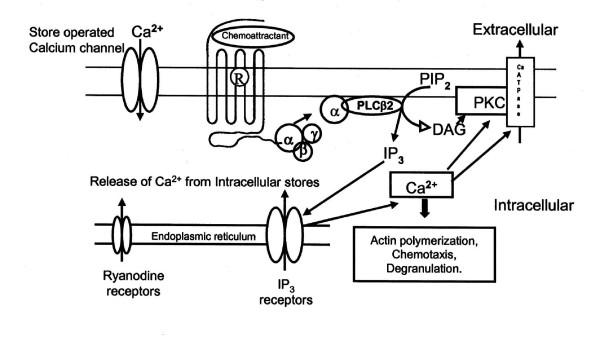
**Cartoon depicting Intracellular Ca^2+ ^signalling network**. Binding of ligand to the seven transmembrane domain receptors activates release of calcium from intracellular stores through IP3 signalling pathway or ryanodine receptors and also initiates influx of calcium through various channels. Increase in Ca^2+ ^results in various cellular responses.

Calcium from the extracellular space enters the cell cytoplasm through various types of channels, i.e. voltage operated Ca^2+ ^channels (VOCCs), ligand-gated non-specific cation channels (LGCCs) and receptor-activated Ca^2+ ^channels (RACCs). Calcium can also be released from internal Ca^2+ ^stores through inositol 1,4,5-triphosphate (IP3) or ryanodine receptors and is replenished by store-operated channels (SOCs) (Fig. 6) [16]. Presence of ryanodine-sensitive calcium stores that might be involved in receptor mediated chemotaxis has been reported in human PMNL [17].

During chemotaxis localised increases in [Ca^2+^]_i _occur. Two distinct [Ca^2+^]_i _storage sites have been identified in PMNL. One site was located peripherally under the plasma membrane and the other in the juxtanuclear space. The central [Ca^2+^]_i _storage site released [Ca^2+^]_i _in response to fMLP, whereas engagement and clustering of CD11b/CD18 integrins caused [Ca^2+^]_i _release from the peripheral stores [18]. The peripheral stores of [Ca^2+^]_i _are known to be regulated by the cytoskeleton interactions [19]. The rise in cytosolic [Ca^2+^]_i _concentration is caused by the mobilization of Ca^2+ ^from intracellular stores to the cytosol followed by an immediate increase in plasma membrane permeability to extracellular calcium. Efflux of [Ca^2+^]_i _is mediated by calmodulin dependent calcium adenosine triphosphatase (Ca-ATPase). Ca-ATPase probably serves as the regulatory and homeostatic mechanism required to maintain low [Ca^2+^]_i _concentrations. When the cytosolic [Ca^2+^]_i _concentration is increased either by fMLP or ionophore, efflux of [Ca^2+^]_i _from the cell occurs as a result of calmodulin mediated activation of Ca-ATPase [20].

Mobilization of Ca^2+ ^is upstream to various biochemical and functional events stimulated in PMNL by various chemoattractants. Therefore, to understand the mechanism of various defects seen in CML PMNL, we have studied mobilization of Ca^2+ ^in response to chemoattractant stimulation. The intracellular free calcium and the total calcium release, i.e. calcium released intracellularly and extracellularly were studied in CML PMNL in comparison to normal PMNL.

Our studies showed altered calcium homeostasis in CML PMNL. The analysis of fluo-3 loaded normal and CML PMNL showed heterogeneity with respect to the basal [Ca^2+^]_i _levels and [Ca^2+^]_i _levels reached after stimulation. On further stimulation of the chemoattractant stimulated PMNL with calcium ionophore, the heterogeneity in [Ca^2+^]_i _levels decreased. Vast heterogeneity was seen in PMNL with respect to the sensitivity of quenching of [Ca^2+^]_i _by EGTA and MnCl_2_. Similar heterogeneity was reported earlier. Elsner et al reported heterogeneity in [Ca^2+^]_i _levels in human PMNL [21]. Additionally, Elsner et al demonstrated heterogeneity in signal transduction pathways leading to mobilization of cytosolic calcium in human PMNL in response to fMLP, C5a and IL-8/NAP-1. Studies by Metzner et al suggested that this heterogeneity could be because individual PMNL required different threshold concentrations of stimulant to induce release of [Ca^2+^]_i _[22]. Yee et al have shown that treatment of PMNL with lipopolysaccharide (LPS) leads to increase in [Ca^2+^]_i _levels and subsequent stimulation with fMLP resulted in further elevation of [Ca^2+^]_i_. LPS augmented the overall responsiveness of a population of PMNL by causing a subpopulation of cells to become highly responsive and able to generate changes in [Ca^2+^]_i _upon low level stimulation [23]. This heterogeneity in PMNL with respect to [Ca^2+^]_i _mediated signal transduction may be important in the fine control of the non-specific immune system in response to weak environmental signals.

The pattern of [Ca^2+^]_i _release on fMLP stimulation differed from that of C5a stimulation. The response to C5a was delayed as compared to that seen for fMLP. This was true for both CML and normal PMNL. This could be because of different time kinetics for internalization and recycling of FPR and C5aR in human PMNL [24]. We have seen that in normal PMNL fMLP receptor internalization occurred by 2–5 min whereas C5a receptor internalization was delayed and occurred by 5–10 min. The reported values of Ca^2+ ^in fluo-3 loaded PMNL are 825 ± 94 nM and 798 ± 102 nM on stimulation with fMLP and C5a respectively [25]. These values are much higher than what we have obtained. These differences in calcium concentrations could be due to the differences in the experimental conditions. Moreover, the levels of calcium reported by Lepidi et al were presumably in the Caucasian population. The differences in calcium levels could be because of the racial differences.  

Ca^2+ ^levels are regulated by the interplay of kinases and phosphatases.  Phosphorylation is reported to down regulate both agonist induced Ca^2+ ^entry and Ca^2+ ^mobilization [26]. Bcr-abl, a chimeric protein expressed by the Philadelphia chromosome has a high and unregulated tyrosine kinase activity. Many different proteins are aberrantly phosphorylated in the cells expressing bcr-abl [27]. Unique phosphatases that can be modulated by α-interferon are also involved in the abnormalities in CML [28]. High phosphotyrosyl phosphatase activity was observed in PMNL from CML patients in the chronic phase. The activity may be characteristic of mature cells and may regulate cellular events through dephosphorylation of p210bcr-abl [29]. Protein tyrosine phosphatases (PTPs)-PTP1B is enhanced in cells expressing p210bcr-abl and has been shown to play a role in dephosphorylation of p210bcr-abl in vivo. PTP1B may function as a specific, negative regulator of p210bcr-abl signalling in vivo. CD45, a family of transmembrane PTPs is expressed in PMNL. Altered expression of CD45 isoforms has been reported in myeloid leukaemias [30]. The altered expression of kinases and phosphatases may alter phosphorylation of various proteins in CML PMNL. Batliwala et al have reported altered phosphorylation pattern of proteins in CML PMNL. In unstimulated CML PMNL, pp1 and pp5 were extensively phosphorylated while phosphorylation of pp3 had reduced as compared to that in normal PMNL. Upon PMA stimulation, normal PMNL showed phosphorylation of pp1 and pp4. But, in contrast to normal PMNL, pp1 and pp4 in CML PMNL did not respond to PMA [31]. Additionally, alterations in the surface proteins and glycoproteins of plasma membrane of CML PMNL have been reported [32,33]. These alterations in various proteins and enzymes may contribute to the altered Ca^2+ ^homeostasis in CML PMNL.

Ras related GTPases – rho, rac and cdc42 regulate polymerization of actin to produce stress fibres and lamellipodia [34]. Expression of bcr-abl affects ras and ras related super family of small GTPases [35,36]. Expression of p210bcr-abl also resulted in reorganization of the actin cytoskeleton in 32DC13 cells [27], suggesting that alteration of rasGTPases probably resulted in alteration of actin network. Altered actin network could affect release of [Ca^2+^]_i _from the intracellular stores and efflux of [Ca^2+^]_i _from the cell cytoplasm. Earlier we have observed that in CML PMNL actin polymerization in response to chemoattractant stimulation was delayed and significantly lower. Moreover, distribution of F-actin in CML PMNL was altered as compared to that in normal PMNL [10,12]. Sachhi et al had reported that PMNL from neonates showed defective chemotaxis, failure in polymerization of actin and lower levels of [Ca^2+^]_i_, following stimulation with fMLP or zymosan activated serum [37]. In PMNL, the peripheral stores of calcium are regulated by the cytoskeletal protein interactions [18]. In turn, increased [Ca^2+^]_i _levels cause fragmentation of F-actin by activation of actin severing and capping proteins such as gelsolin and macrophage capping protein [2]. In view of this, the lower basal levels of [Ca^2+^]_i _and the increased extent of [Ca^2+^]_i _release seen in CML PMNL in response to chemoattractants could be because of altered regulation of [Ca^2+^]_i _stores by altered actin network.

Mutations or functional abnormalities in the various Ca^2+ ^transporters lead to plethora of diseases. In skeletal muscle pathology, mutations in ryanodine receptors cause malignant hyperthermia and porcine stress syndrome. Mutations in calcium pump cause Brody disease. Various abnormalities in calcium handling proteins have been reported in the heart during aging, hypertrophy, diabetes, etc. [38]. Alterations in [Ca^2+^]_i _levels of PMNL are associated with various disorders. Lower [Ca^2+^]_i _levels were observed in PMNL from asthmatic patients, in response to PAF stimulation [39]. These lower levels of [Ca^2+^]_i _were due to low influx of Ca^2+^. In localized juvenile periodontitis (JP), the basal levels of [Ca^2+^]_i _in PMNL were comparable to that of normal. But, fMLP and C5a induced [Ca^2+^]_i _levels were reduced in JP. This was due to decreased Ca^2+ ^influx. These reduced [Ca^2+^]_i _levels were associated with reduced PKC levels and defective chemotaxis [40,41]. Similarly in aged individuals, PMNL showed low [Ca^2+^]_i _levels that were associated with defective chemotaxis and oxidative burst [42]. Thus, lower [Ca^2+^]_i _levels were often associated with altered chemotaxis. In contrast to this, calcium transients were not required for other PMNL functions such as an oxidative burst. For example, PMNL from Myelodisplastic syndrome (MDS) patients are defective in respiratory burst. Studies by Nakaseko et al showed that basal and fMLP stimulated [Ca^2+^]_i _levels in MDS PMNL were comparable to those in normal PMNL [43]. However, O'Flaherty et al have reported that [Ca^2+^]_i _transients potentiated PMNL degranulation and respiratory burst [15]. [Ca^2+^]_i _is also known to regulate chemoattractant receptor availability in PMNL. In PMNL from asthmatic patients, lower [Ca^2+^]_i _levels were associated with lower PAF receptor [39]. O'Flaherty et al have reported that [Ca^2+^]_i _depleted PMNL showed decrease in receptors for fMLP, LTB4 and PAF [15]. Our studies showed that though not statistically significant, levels of [Ca^2+^]_i _were lower in unstimulated CML PMNL as compared to normal PMNL. These [Ca^2+^]_i _levels in CML PMNL were lower up to 1 min after stimulation with fMLP and C5a. However, the total calcium release was more in CML PMNL than that in normal PMNL. This suggested that efflux of [Ca^2+^]_i _was more and faster in CML PMNL than that in normal PMNL. Earlier we have shown that CML PMNL showed a lower number of FPR, defects in actin and tubulin polymerization, pinocytosis and degranulation. But, stimulation of oxidative burst in CML PMNL was comparable to that in normal PMNL. Thus, our observations are in agreement with earlier reports that [Ca^2+^]_i _levels regulate availability of chemoattractant receptors and low [Ca^2+^]_i _levels are associated with defects in actin polymerization, chemotaxis and degranulation. However, [Ca^2+^]_i _transients are not essential for stimulation of an oxidative burst. Additionally our results suggest that the levels of [Ca^2+^]_i _maintained in the cell immediately after stimulation and not the amount of total calcium released are important in stimulation of various functional events in PMNL. PKC is one of the important messengers leading to various functional events in PMNL. In signal transduction pathway, PKC is downstream to Ca^2+ ^mobilization. In CML PMNL, expression of calcium dependent PKC isoform α was lowered [44]. Various functional events in PMNL are mediated partly or totally through PKC. Therefore, it could be that the defects in various functional events in CML PMNL are due to alterations in PKC caused by altered Ca^2+ ^homeostasis or are the direct effect of altered Ca^2+ ^homeostasis per se.

## Conclusion

In summary, our studies show that Ca^2+ ^homeostasis in CML PMNL is altered. This could be one of the contributing factors for the reduced responses seen in CML PMNL. Further studies of the calcium oscillations in CML PMNL and the Ca-ATPase would help in pin pointing the defects in calcium homeostasis in CML PMNL.

## Methods

### Reagents

Ficoll-Hypaque, 4-(2-hydroxyethyl)-1-piperazine ethane sulphonic acid (HEPES), ethyleneglycol-bis-(3-aminoethyl)-tetra acetate (EGTA), manganese chloride (MnCl_2_), n-formyl-methionyl-leucyl-phenylalanine (fMLP), complement factor 5a (C5a) and Calcium ionophore A23187 were from Sigma. Dimethyl sulfoxide (DMSO) was from Seezle, Germany. Fluo-3-pentapotassium salt and fluo-3AM – the pentaacetoxymethyl ester of fluo-3 and calcium calibration kit were from Molecular Probes (Eugene, USA).

### Patients

Patients were diagnosed for CML on the basis of standard clinical and haematological criteria. Heparinized peripheral blood was collected from CML patients in chronic phase of the disease, before commencement of therapy. Healthy individuals were used as controls.

### Isolation of PMNL

PMNL from peripheral blood were isolated on a ficoll-hypaque gradient [45]. To lyse RBCs the cell pellet was given a hypotonic shock using 3 parts of chilled Milli-Q water. Isotonicity was restored by the addition of 1/3^rd ^volume of 0.6 M KCl. The PMNL were pelleted and suspended in phosphate buffered saline (PBS). Viability of cells was more than 99% as checked by the erythrocin B dye exclusion test. The PMNL fraction was 95% enriched as checked by Giemsa's staining.

### Fluo-3 loading

One millimolar stock solution of fluo-3AM was prepared in dry DMSO. Fluo-3 loading of PMNL was carried out as described by Vandenberghe et al with some modifications [46]. Briefly, PMNL were suspended at a density of 1× 10^7 ^cells per ml in fluo-3 loading buffer (10 mM HEPES, 137 mM NaCl, 5 mM KCl, 1 mM Na_2_HPO_4_, 5 mM glucose and 0.5 mM MgCl_2_, pH 7.4) containing 5 μM fluo-3AM. PMNL were incubated for 30 min at 37°C with gentle agitation. Cells were then washed twice with Ca^2+ ^estimation buffer (i.e. fluo-3 loading buffer with 1 mM CaCl_2_) to remove extra cellular fluo-3AM. Finally the cell density was adjusted to 1 × 10^7 ^cells per ml in the calcium estimation buffer. The cells were kept in dark at 4°C till use.

### Measurement of [Ca^2+^]_i _by flow cytometry

10^6 ^PMNL were suspended in one ml Ca^2+ ^estimation buffer and incubated in a 37°C water bath, for 5 min before each assay. The samples were run on FACScalibur (Becton-Dickinson, USA) using Cell-Quest software. The samples were excited by an argon ion laser at 488 nm and emission was measured at 525 nm on a logarithmic scale. Before addition of the stimuli, fluorescence of the fluo-3 loaded PMNL was measured. Optimum stimulation of PMNL in suspension with fMLP was seen at a concentration of 10^-8 ^M [10,11] whereas with C5a it was seen at 10 fold lower concentration [47]. Therefore, PMNL were stimulated with 10^-8 ^M fMLP and 10^-9 ^M C5a. Fluorescence was estimated at 10 sec, 30 sec and 60 sec after addition of the stimuli. For each acquisition, minimum 5000 events were collected. F_max _was estimated using PMNL treated with calcium ionophore A23187. Quenching of external and internal fluo-3 fluorescence was estimated using 0.4 M EGTA and 2 mM MnCl_2_, respectively. Median channel number was taken as a measure of fluorescence intensity of the sample.

### Calibration procedure

To convert the arbitrary fluorescence units of fluorescence measured by spectrofluorimeter into absolute Ca^2+^, a calibration procedure described by Vandenberghe was used [46]. The dissociation constant, i.e. Kd for Ca^2+ ^bound fluo-3 was calculated using calibration kit. The maximum concentration of Ca^2+ ^in the kit, i.e. 39.8 μM was taken as F_max _and buffer without Ca^2+ ^was taken as F_min_. The calcium bound fluo-3 and free fluo-3 both showed excitation and emission peak at 506 nm and 526 nm, respectively. Therefore, Kd of fluo-3 was calculated by measuring fluorescence of fluo-3 pentapotassium salt (5 μM) using these excitation and emission wavelengths on a Shimadzu RF1501 spectrofluorimeter. The cuvette holder was maintained at 37°C.

The Kd of fluo-3 was calculated using the equation:

Kd = [Ca^2+^]_i_/[(F-F_min_)/(F_max_-F)]     equation I [48]

The experiment was done four times independently.

### Measurement of total Ca^2+ ^by spectrofluorimetry

10^6 ^fluo-3 loaded PMNL were suspended in two ml Ca^2+ ^estimation buffer and incubated in a water bath at 37°C for 5 min, before each assay. PMNL were then stimulated with 10^-8 ^M fMLP or 10^-9 ^M C5a. The fluorimetric reading was taken before stimulation and at 10 sec, 30 sec and 60 sec after stimulation.

Autofluorescence was calculated using unloaded PMNL. F_max_, the maximum fluorescence was obtained by treating the cells with 10 μM of calcium ionophore A23187. F represents the fluorescence of the test sample. Quenching of external and internal fluo-3 fluorescence was estimated using 0.4 M EGTA and 2 mM MnCl_2_, respectively. Fluo-3-Mn^2+ ^complex is eight times less fluorescent as compared to fluo-3-Ca^2+ ^complex. Under experimental conditions, the quenching of fluo-3 by MnCl_2 _was low as compared to autofluorescence of PMNL, hence autofluorescence was considered as F_min _[49]. The spectrofluorimetric values were converted to concentration of Ca^2+ ^by using equation I.

### Statistical analysis

Non-parametric tests were applied for statistical analysis of the data. Wilcoxon signed rank test was used to compare the median fluorescence channel and absolute calcium concentrations within normal and CML samples. The Mann-Whitney Wilcoxon test was used to compare the median fluorescence channel and absolute calcium concentrations of normal and CML samples. The Mann-Whitney Wilcoxon test was also used to compare the median fluorescence channel and absolute calcium concentration values of fMLP stimulated PMNL with that of C5a stimulated PMNL.

## Competing interests

The author(s) declare that they have no competing interests.

## Authors' contributions

CMR participated in the standardization of the techniques, acquisition of the data, analysis and drafting the manuscript. SHA was a clinical collaborator and was involved in diagnosis of the patients and providing clinical samples for the studies. NRN conceived the study, participated in the design of the study, standardization of the techniques, acquisition of the flow cytometry data, coordination and helped to draft the manuscript. All the authors have read and approved the final manuscript.
